# Complete Genome Sequence of the Myoviral Bacteriophage YS35, Which Causes the Lysis of a Multidrug-Resistant *Pseudomonas aeruginosa* Strain

**DOI:** 10.1128/genomeA.01395-17

**Published:** 2018-02-08

**Authors:** Sheng Yu, Honglan Huang, Yuchong Hao, Hongyan Shi, Chunyan Zhao, Yanbo Sun

**Affiliations:** a Department of Pathogen Biology, College of Basic Medical Sciences, Jilin University, Changchun, People’s Republic of China

## Abstract

The lytic bacteriophage YS35, which is capable of lysing multidrug-resistant *Pseudomonas aeruginosa* strains, was isolated from a sewage sample. Here, we describe the complete genome sequence of this myoviral bacteriophage, which contains 93,296 bp of double-stranded DNA and has a 49.4% G+C content.

## GENOME ANNOUNCEMENT

*Pseudomonas aeruginosa* strains are glucose-nonfermenting Gram-negative opportunistic pathogens that cause health care-associated infections (1). Moreover, the growing prevalence of multidrug-resistant (MDR) and extensively drug-resistant (XDR) *P. aeruginosa* strains is considered a tremendous threat to global public health (2, 3). Recently, the application of phage-based products to treat *P. aeruginosa* infections *in vitro* and *in vivo* has been reported (4). We isolated the lytic bacteriophage YS35 from a sewage sample collected at the First Affiliated Hospital of Jilin University, China, using the MDR *P. aeruginosa* strain DH35 isolated from a patient with chronic pulmonary infection. The bacteriophage YS35 belongs to the *Myoviridae* family based on the morphological features determined using transmission electron microscopy.

Genomic DNA of phage YS35 was extracted from purified phage stock using a phenol extraction method (5). Whole-genome sequencing was conducted using the Illumina HiSeq 4000 platform at Hangzhou Lianchuan Biotechnology Co., Ltd. (Hangzhou, China). Quality-controlled trimmed reads were assembled with MITObim version 1.8 software. The coverage was on the order of 1,000×, and one contig was obtained. Potential open reading frames (ORFs) were identified with GeneMarkS version 4.17 (6), and the functions of the ORFs were predicted using BLASTp searches against the nonredundant NCBI database. tRNAscan-SE version 1.3.1 (7) was employed to predict tRNAs.

The YS35 genome comprises 93,296 bp of linear double-stranded DNA with a G+C content of 49.3% and 13 tRNAs. In total, 172 ORFs were predicted, only 28 of which had a putative function. Furthermore, 52 ORFs are rightward oriented, while 120 ORFs are leftward oriented. According to a BLASTn analysis, the nearest neighbors (query coverage range from 95% to 97%, identity range from 98% to 99%) of phage YS35 are *Pseudomonas* phages vB_PaeM C2-10 Ab02, phage vB_PaeM C2-10 Ab10, phage vB_PaeM C2-10 Ab1, phage vB_PaeM C2-10 Ab8, and phage vB_PaeM C2-10 Ab15. These five phages, which were isolated in Abidjan, Côte d’Ivoire, are similar to PAK_P1, which was isolated in France. Therefore, the phage YS35 is closely associated with PAK_P1-like virus.

The comprehensive analysis of the genes and proteins encoded by phage YS35 can provide new insight into phage-bacteria coevolution and strategies for the therapeutic use of bacteriophages.

### Accession number(s).

The complete genome sequence of bacteriophage YS35 has been deposited in GenBank under accession number MF974178.
